# A new method based on quiet stance baseline is more effective in identifying freezing in Parkinson's disease

**DOI:** 10.1371/journal.pone.0207945

**Published:** 2018-11-26

**Authors:** Hiram Cantú, Julie N. Côté, Julie Nantel

**Affiliations:** 1 Departamento de Ingeniería Biomédica, Vicerrectoría de Ciencias de la Salud, Universidad de Monterrey, San Pedro Garza García, Nuevo León, México; 2 Occupational Biomechanics and Ergonomics Laboratory, Michael Feil and Ted Oberfeld/CRIR Research Centre, Jewish Rehabilitation Hospital, Laval, QC, Canada; 3 Department of Kinesiology and Physical Education, McGill University, Montréal, QC, Canada; 4 School of Human Kinetics, University of Ottawa, Ottawa, ON, Canada; University of Catania, ITALY

## Abstract

Freezing, an episodic movement breakdown that goes from disrupted gait patterns to complete arrest, is a disabling symptom in Parkinson’s disease. Several efforts have been made to objectively identify freezing episodes (FEs), although a standardized methodology to discriminate freezing from normal movement is lacking. Novel mathematical approaches that provide information in the temporal and frequency domains, such as the continuous wavelet transform, have demonstrated promising results detecting freezing, although still with limited effectiveness. We aimed to determine whether a computerized algorithm using the continuous wavelet transform based on baseline (i.e. no movement) rather than on amplitude decrease is more effective detecting freezing. Twenty-six individuals with Parkinson’s disease performed two trials of a repetitive stepping-in-place task while they were filmed by a video camera and tracked by a motion capture system. The number of FEs and their total duration were determined from a visual inspection of the videos and from three different computed algorithms. Differences in the number and total duration of the FEs between the video inspection and each of the three methods were obtained. The accuracy to identify the time of occurrence of a FE by each method was also calculated. A significant effect of Method was found for the number (*p* = 0.016) and total duration (*p* = 0.013) of the FEs, with the method based on baseline being the closest one to the values reported from the videos. Moreover, the same method was the most accurate in detecting the time of occurrence, and the one reaching the highest sensitivity (88.2%). Findings suggest that threshold detection methods based on baseline and movement amplitude decreases capture different characteristics of Parkinsonian gait, with the first one being more effective at detecting FEs. Moreover, robust approaches that consider both time and frequency characteristics are more sensitive in identifying freezing.

## Introduction

Along the cardinal symptoms in Parkinson’s disease (PD) of bradykinesia, tremor, rigidity and postural instability, some individuals with PD develop episodic motor breakdowns that are more common in advanced stages of the disease and are known as freezing. Although freezing has been reported in different tasks involving lower limbs [1–8], upper limbs [7–13], and even speech [14–17], it is more commonly studied in gait (freezing of gait, FOG) [18–27] probably due to its consequences on functional mobility and falls. FOG is defined as a ‘brief, episodic absence or marked reduction of forward progression of the feet despite the intention to walk’ [26]. As suggested by Vercruysse and colleagues in a recent review [28], this wide definition covers from disrupted gait patterns to complete gait movement arrests. Thus, it is not surprising that there is also a wide amount of methods and approaches to identify FOG while testing in a laboratory and/or during daily life activities.

Currently there is only one tool validated to identify FOG, which consists in a self-assessment questionnaire in which individuals with PD report how often they experience freezing [29, 30]. This tool has been shown to be highly sensitive in identifying those individuals with PD who experience freezing (freezers) from those who do not experience freezing (non-freezers). However, the Freezing of Gait Questionnaire (FOGQ) does not help to quantify the specific time where this phenomenon occurs.

In an attempt to study freezing in lower limb and upper limb of individuals with PD, several objective approaches that fit with the definition of freezing have been used. In a series of experiments studying movement arrests in PD while navigating in a virtual reality scenario, freezing was defined as a between-footstep latency that exceeded twice the patients’ modal footstep latency [2, 3, 31]. Ehgoetz Martens and colleagues [19] investigated freezing in PD while walking through a doorway in a variety of conditions and defined FOG as those periods where gait velocity of participants was between a complete stop and one standard deviation of their regular velocity above zero. Freezing has been also assessed in terms of kinetic data [4, 32]. Nantel and colleagues [4] tested a group of individuals with PD who experienced freezing while raising the legs alternately, and by measuring the vertical forces acting under the feet with a force plate, they were able to quantify freezing. In their study, a freezing episode (FE) was defined as an interval duration between cycles larger than 1.2 times the average duration of the intervals of the three previous cycles or at least twice the average duration interval in the whole trial where participants were unable to lift their feet from the floor.

Despite the different approaches described above, complete movement arrests and initial amplitude decreases are the most common criteria used to identify freezing in PD. In their studies, Almeida’s group [9, 10] defined freezing as any period of time of at least 1 s where no movement was displayed, as no change in the amplitude, when patients with PD performed an upper limb sliding task. In line with this method, a period lasting more than 1 s with no successful movement displayed in any limb preceded by amplitude decreases and increased movement frequency was used to investigate freezing in a repetitive drawing task [12]. However, several studies from a different group have considered freezing as periods of severely disrupted movement that included a decrease of 50% of the initial amplitude as the beginning of the motor arrest [8, 11, 33]. This wide diversity of methodologies and criteria to identify freezing in PD raise the need of a standardized method to ensure that the same phenomenon is being studied and that allows comparisons across studies.

Recent efforts have been made to identify freezing by using robust mathematical functions. Bächlin and colleagues [34] used the fast Fourier transform (FFT) to analyze motor arrests in individuals with PD while performing basic walking tasks that represented daily activities. The FFT was computed on 3D acceleration data obtained from inertial measurement units (IMUs) attached to the legs at the shank and the thigh, reaching acceptable sensitivity and specificity (73.1% and 81.6%, respectively). Unlike the FFT that provides information only in the frequency domain, the continuous wavelet transform (CWT) decomposes a signal into wavelets that are not only scaled in the frequency domain but also shifted in the time domain [35, 36]. This time and frequency decomposition requires a ‘mother wavelet’ as a source from which wavelets are constructed. In a recent study investigating the occurrence of freezing while walking and turning, CWT with the use of Daubechies wavelets of 4th order as the ‘mother wavelet’ was applied to accelerations obtained from an IMU placed on the patients’ shank. The use of CWT showed a better discrimination of freezing from normal walking, as well as larger values for sensitivity (84.9%) and specificity (81.01%) in identifying freezing and normal movement, respectively [37], compared to the use of FFT [34]. This suggests that freezing, being a complex phenomenon, requires the use of methods that consider time and frequency domains equally in order to robustly detect motor arrests in individuals with PD. However, the method presented in [37] involves the analysis of accelerations obtained from IMUs. This poses the disadvantage of having imperfections that include external signal noise, signal filtering errors, and integration drift. This might lead to lower accuracy compared to that in laboratory tracking systems, which could notably have a significant impact when studying gait impairments [38, 39].

Therefore, the aim of this study was to determine an effective method to identify freezing in individuals with PD who experience this symptom while performing a rhythmic stepping-in-place (SIP) task [4, 5]. We sought to determine whether a method novel based on a recently described set of robust mathematical approaches that decompose a signal considering the time and frequency domains [37], but applied to position data obtained from a laboratory tracking system and based on baseline (i.e. no movement) data, better discriminates FEs from normal movement, in terms of the number of occurrence, the duration, and the time of occurrence compared to a method based on amplitude decreases. We hypothesized that a method based on baseline data would better capture when motor arrest occur since amplitude decreases not only would include motor arrest but also abnormal gait patterns.

## Materials and methods

Ethics approval was received from the Research Ethics Board of the Centre for Interdisciplinary Research in Rehabilitation (CRIR) of Greater Montreal, and all participants gave written informed consent.

### Participants

A group of twenty-six patients with PD were recruited from the Cummings Centre for Seniors in Montreal, Quebec, and from the Quebec Parkinson Network to participate in this study. All participants performed the experimental protocol twice on separate days with a mean of 8.3 ± 3.7 days between both sessions. For the first session individuals with PD were on dopaminergic medication while for the second session, they had withdrawn from medication for at least the previous 12 hours. Both sessions were conducted at the same time of the day and under the same conditions to avoid within-day motor function fluctuations. Participants were excluded from this study if: (1) they had any neurological, orthopedic, or muscular disorder other than PD, (2) they had a mild cognitive impairment based on a score below 26 on the Montreal Cognitive Assessment (MoCA), (3) they had undergone deep brain stimulation surgery, (4) they were taking medication affecting balance, and/or (5) they had a history of diabetes. All participants indicated their informed consent by signing forms approved by the Research Ethics Board of the Centre for Interdisciplinary Research in Rehabilitation (CRIR) of Greater Montreal.

### Clinical evaluation

Prior to their participation in the first session, all participants were clinically assessed by the same examiners with five different evaluations in their on-medication state: (1) a medical history questionnaire, (2) the Montreal Cognitive Assessment (MoCA) test, (3) the Unified Parkinson Disease Rating Scale Part 3 (UPDRS-III), (4) the Hoehn and Yahr (H&Y) scale, and (5) the Freezing of Gait Questionnaire (FOGQ). The H&Y scale and the UPDRS-III were repeated in the second session to assess participants in their off-medication state. Participants were identified as freezers (*n* = 12) if freezing was observed and/or they answered experiencing at least once a month (score of 1 or greater) to the FOGQ question #3: *“do you feel that your feet get glued to the floor while walking*, *making a turn or when trying to initiate walking*.*”*

### Experimental protocol

All participants performed a repetitive SIP task during both sessions, a task that has been previously used to assess freezing in individuals with PD [4]. The task consists of alternately raising the legs in a standing position and at a self-selected pace that was chosen from two practice trials of 20 s where participants were instructed to perform the task as fast and as comfortably as possible. The experimental protocol included two trials of the SIP task, the first of 30 s and the second of 120 s duration. Before starting the task, participants were provided with a 10-beat auditory feedback from a metronome set at the self-selected pace obtained from the practice trials and they were asked to memorize and perform the task following this rhythm. Subjects were questioned concerning fatigue and allowed to rest in between trials if needed. While performing the trials, participants were secured in a harness attached to the ceiling that did not restrict natural movement but prevented large body sway and falls.

Video data was obtained by filming each session of the experimental protocol with a video camera placed in the frontal and sagittal plane about 5 m away from participants. Kinematic data was recorded during the whole trials and 5s before initiating the SIP task in order to have a quiet stance baseline. Anthropometric measures were taken at the end of the first session. Initial foot placements were replicated in each participant’s second session.

### Data acquisition

A six high-resolution camera, Vicon MX3 motion capture system (VICON, Oxford Metrics Ltd., Oxford, UK) was used to record the positions and movements of both feet with a sampling frequency of 100 Hz. A set of 8 passive and reflective markers was fixed to the body using double-sided adhesive tape on the following anatomical landmarks of the feet: heel (on the calcaneous), ankle (on the lateral malleolus), fifth metatarsal, and first distal phalanx, all of them bilateral.

### Data analysis

A FE was defined as a minimum time of 0.5 s where participants were unable to completely lift a foot from the floor during the SIP task. A pair of trained observers visually and independently inspected the videos filmed for each participant in both sessions to visually detect all trials in which freezing occurs. Then they identified the number of FEs and determined their duration time. If there was a disagreement between observers, videos were sent back to each of them for review. Videos were cropped in different files to assure that observers’ inspection was made only over each of the SIP task times.

The left heel (LHEE) and right heel (RHEE) markers were chosen for further analysis in this study in order to identify FEs considering that these markers are the first ones to lift from the floor when performing the SIP task. The coordinates of LHEE and RHEE were obtained in the x-axis for anterior-posterior direction, in the y-axis for medial-lateral direction, and in the z-axis for superior-inferior direction.

Automated detection of FEs was performed decomposing in wavelets the superior-inferior data of LHEE and RHEE, and merging them together, using a CWT with the form of 4^th^ order Daubechies mother wavelet in three different methods and algorithms that were computed in Matlab (MathWorks, Massachusetts, USA). All three methods calculated the number and duration time of FEs. The main differences between the three methods used in the computations rely first on the conditions used to define thresholds in order to discriminate freezing from normal movement and second on unique freezing criteria, described as follows:

- Method 1 (M1). 65 standard deviations (SD) of the first 5 s of quiet stance were computed on the decomposed wavelets of LHEE and RHEE in order to define the threshold to discriminate normal walking from a FE in each marker since it was the amount of SD with better results. A final signal was created by merging the decomposed wavelets of LHEE and RHEE and by considering as a FE those data points where both markers were below their own threshold. The number and duration of FEs were obtained by identifying those set of data lasting a minimum of 0.5 s below the threshold. Moreover, a window of 2 s was considered the minimum time to overcome a FE. In other words, if a set of data was located in between two FEs and lasted less than 2 s, it was considered as part of the same FE.
- Method 2 (M2). Same methodology than M1 but without considering the 2 s window to overcome a FE.
- Method 3 (M3). Same methodology than M1, with a minimum FE time duration of 0.5 s and a minimum of 2 s to overcome a FE, but computing a reduction to 25% or less of the initial amplitude as the threshold to identify FEs instead of baseline.

The 2 s window criteria to overcome a FE was also considered for the number and total duration times obtained from the video inspection that were further compared to M1 and M3 (video 1, V1, and video 3, V3, respectively), but not for the video that was compared to M2 (video 2, V2). The absolute value of the differences in the number and total duration time of the FEs between the video inspection and each of the methods (V1 vs. M1, V2 vs. M2, and V3 vs. M3) were calculated.

**Fig 1:**
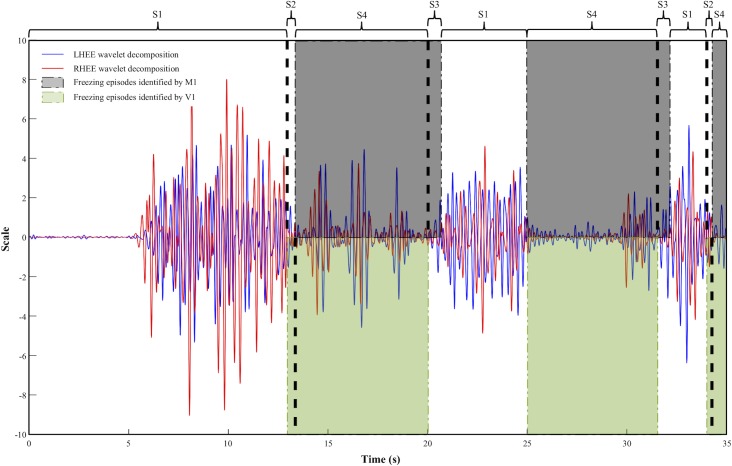
Graphic representation of freezing detection by video inspection and a computerized algorithm. A representative thirty-five second trial sample (5 s of baseline and 30 s of the stepping-in- place task) of the wavelet decomposition by the continuous wavelet transform of both heels, left (LHEE, blue line) and right (RHEE, red line). The gray windows on the top of the figure represent the freezing episodes identified by the algorithm of Method 1 (M1); the green windows at the bottom of the figure represent the freezing episodes identified by the observers in the Video 1 (V1). S1 represents the time where both, video and method, do not identify a freezing episode (FE), S2 represents the time where a FE was identified only in the video, S3 the time where a FE was identified only by the method, and S4 represents the time where both, video and method, identify a FE.

When comparing data point by data point between video and method, regardless of the number of video and method, four different scenarios were possible:

1. Both, video and method, identifying normal movement.
2. Video identifying a FE while method identifies normal movement.
3. Method identifying a FE while video identifies normal movement.
4. Both, video and method, identifying a FE (Fig 1).

Accuracy to identify the time of occurrence of a FE was calculated by the probability that both video and method identify a FE among all the scenarios where a FE is identified. In other words, the number of times that scenario 4 occurs, divided by the occurrence of scenarios 2, 3 and 4. In order to calculate this, the method data was down-sampled to 2 Hz in order to fit the video data since FEs lasting as low as 0.5 s were reported from the videos.

### Statistical analysis

For all statistical analyses, significance was set to *α* < 0.05. Descriptive statistics of participants were computed (group mean and SD measures). Inter-rater reliability was assessed using Cohen’s kappa for the rating of the number and duration of FEs using the values reported by each observer prior to reaching consensus. Tests of normality were run for all outcome variables. In the case of abnormal distribution, non-parametric statistics were computed. The differences of number and total duration of FEs between the videos and each of the three methods were compared by means of a non-parametric repeated measures Friedman test for equal sample sizes. The accuracy to identify time of occurrence of a FE between the three methods was compared using a non-parametric repeated measures Cochran's Q test for unequal sample sizes. If significant differences were found in non-parametric tests, Wilcoxon post-hoc comparisons were performed to determine pair-wise differences with Bonferroni corrections (setting the ‘alpha’ (*α*) level for significance to 0.05 divided by the number of pairwise comparisons, e.g. 0.05/3 = 0.017). The accuracy of each of the three methods to identify FEs was described by the area under the receiver operating characteristic (ROC) curve and in terms of sensitivity and specificity. For this the comparisons between each of the data points of the video and each of the data points of the methods were used to define sensitivity (both, video and method, identifying a FE) and specificity (both, video and method, identifying normal movement).

## Results

Twelve participants were screened as freezers (10 males, 2 females) and fourteen as non-freezers (9 males, 5 females). Freezers’ mean demographic characteristics and clinical evaluation results are reported in Table 1. Prior to consensus, Cohen’s kappa analysis showed a high degree of agreement between the observers’ reports of the number and duration of FEs within each trial (*k* = 0.961, *p* < 0.001). Based on observers visual inspection, FEs were identified in a total of ten trials in the freezers-group and were further analyzed by the algorithms of the three methods computed. No FEs were found in the trials performed by the non-freezers group.

**Table 1:** Participants’ characteristics and clinical evaluation results (mean (SD)).

Participants characteristic	Freezers (n = 12)	Non-freezers (n = 14)
Age (years)	69.1 (5.7)	66.1 (7.0)
Sex (m/f)	10/2	9/5
MoCA (units)	27.7 (1.4)	28.1 (1.6)
UPDRS-III (on)	28.9 (9.8)	21.7 (8.2)
UPDRS-III (off)	37.9 (9.9)	30.0 (10.7)
H&Y (on)	2.7 (0.9)	1.9 (0.7)
H&Y (off)	3.1 (1.0)	2.3 (0.7)
FOGQ (units)	10.3 (3.5)	0.9 (1.4)
Disease Duration (years)	11.9 (5.3)	3.9 (2.3)

Note: SD: standard deviation; m/f: male/female; MoCA: Montreal Cognitive Assessment; UPDRS-III: Unified Parkinson’s Disease Rating Scale Part 3; on: on medication; off: off medication; H&Y: Hoehn and Yahr; FOGQ: Freezing of Gait Questionnaire.

Table 2 summarizes the differences in number and total duration time of FEs between the videos and methods. There was a statistically significant effect of Method on differences in the number of FEs identified in the ten trials compared to those identified in the videos, (*χ*^2^(2) = 8.267, *p* = 0.016). Mean differences in the number of FEs identified between video and method demonstrated M1 to be the closest one (0.7) followed by M3 and M2 (1.4 and 9.4, respectively) (Table 2). Since three different methods are tested in this analysis, there are three possible pairwise comparisons (V1-MI vs. V2-M2, V1-MI vs. V3-M3, V2-M2 vs. V3-M3); therefore post-hoc analyses with Wilcoxon signed-rank tests were conducted using Bonferroni adjusted *α* level of 0.017 to test each individual hypothesis (0.05/3). Only *p* values below the adjusted *α* are considered statistically significant. There were no significant differences in any pairwise comparison, although a trend towards statistical significance was found between M1 and M2 (*Z* = -2.371, *p* = 0.018).

**Table 2:** Differences in the number and total duration time of freezing episodes (FEs) for each trial. Absolute values of video—method differences are reported.

Trial #	Video 1 –Method 1		Video 2 –Method 2		Video 3 –Method 3	
	Difference Number FEs	Difference Duration FEs (s)	Difference Number FEs	Difference Duration FEs (s)	Difference Number FEs	Difference Duration FEs (s)
1	0	0.17	2	2.09	0	2.19
2	1	4.08	40	8.74	3	3.39
3	0	0.8	3	1.34	0	0.82
4	1	1.88	23	5.19	3	20.8
5	1	0.15	1	0.62	1	2.89
6	2	4.14	20	5.27	0	15.91
7	1	1.7	3	12.49	3	8.2
8	0	0.69	1	1.01	1	0.57
9	0	0.05	0	0.05	1	1
10	1	0.04	1	0.99	2	3.5
Mean (SD)	0.7 (0.7)*	1.4 (1.6)^#^	9.4 (13.6)*	3.8 (4.1)^#^	1.4 (1.3)	5.9 (7.0)

* Trend to significance between video 1 –method 1 and video 2 –method 2 (*p* = 0.018)

^*#*^ Significant difference between video 1 –method 1 and video 2 –method 2 (*p* < 0.01)

A significant effect of Method was found in the differences between video and method for the total duration time of FEs (*χ*^2^(2) = 8.667, *p* = 0.013). Again, since three individual hypotheses are tested in post-hoc Wilcoxon analysis (V1-MI vs. V2-M2, V1-MI vs. V3-M3, V2-M2 vs. V3-M3), significance level was adjusted with Bonferroni correction (*α* = 0.05/3 = 0.017) showing no significant differences between M1 and M3 (*Z* = -2.293, *p* = 0.022) and between M2 and M3 (*Z* = -0.764, *p* = 0.445). However, there was a statistically significant difference in the similitude of the total duration time of FEs between V1 and M1 compared to V2 and M2 (*Z* = -2.666, *p* = 0.008).

Cochran’s Q test indicated significant differences in the accuracy to identify the time of occurrence of FEs between the three methods (*χ*^2^(2) = 148.112, *p* < 0.001). Post-hoc Wilcoxon analysis with Bonferroni correction demonstrated significant differences in all possible pairwise comparisons (see Table 3). M1 reached a 77% accuracy to identify the time occurrence of FEs compared to V1, followed by M3 and M2 (67.8% and 56.1%, respectively).

**Table 3:** Accuracy to identify time of occurrence of a freezing episode (FE) by each method. Scenario 2 represents the time where a FE was identified only in the video, scenario 3 the time where a FE was identified only by the method, and scenario 4 the time where both, video and method, identify a FE. Accuracy is represented by the probability of occurrence of scenario 4 among scenarios 2, 3, and 4.

Method	Scenario 2		Scenario 3		Scenario 4	
	Number of Occurrence	Percentage of Occurrence (%)	Number of Occurrence	Percentage of Occurrence (%)	Number of Occurrence	Percentage of Occurrence (%)
1	63	10.3	78	12.7	472	77*^#^
2	143	26.7	92	17.2	300	56.1*^§^
3	150	26.4	33	5.8	385	67.8^#^^§^

* Significant difference between method 1 and method 2 (*p* < 0.001)

^*#*^ Significant difference between method 1 and method 3 (*p* < 0.001)

^*§*^ Significant difference between method 2 and method 3 (*p* < 0.001)

**Fig 2:**
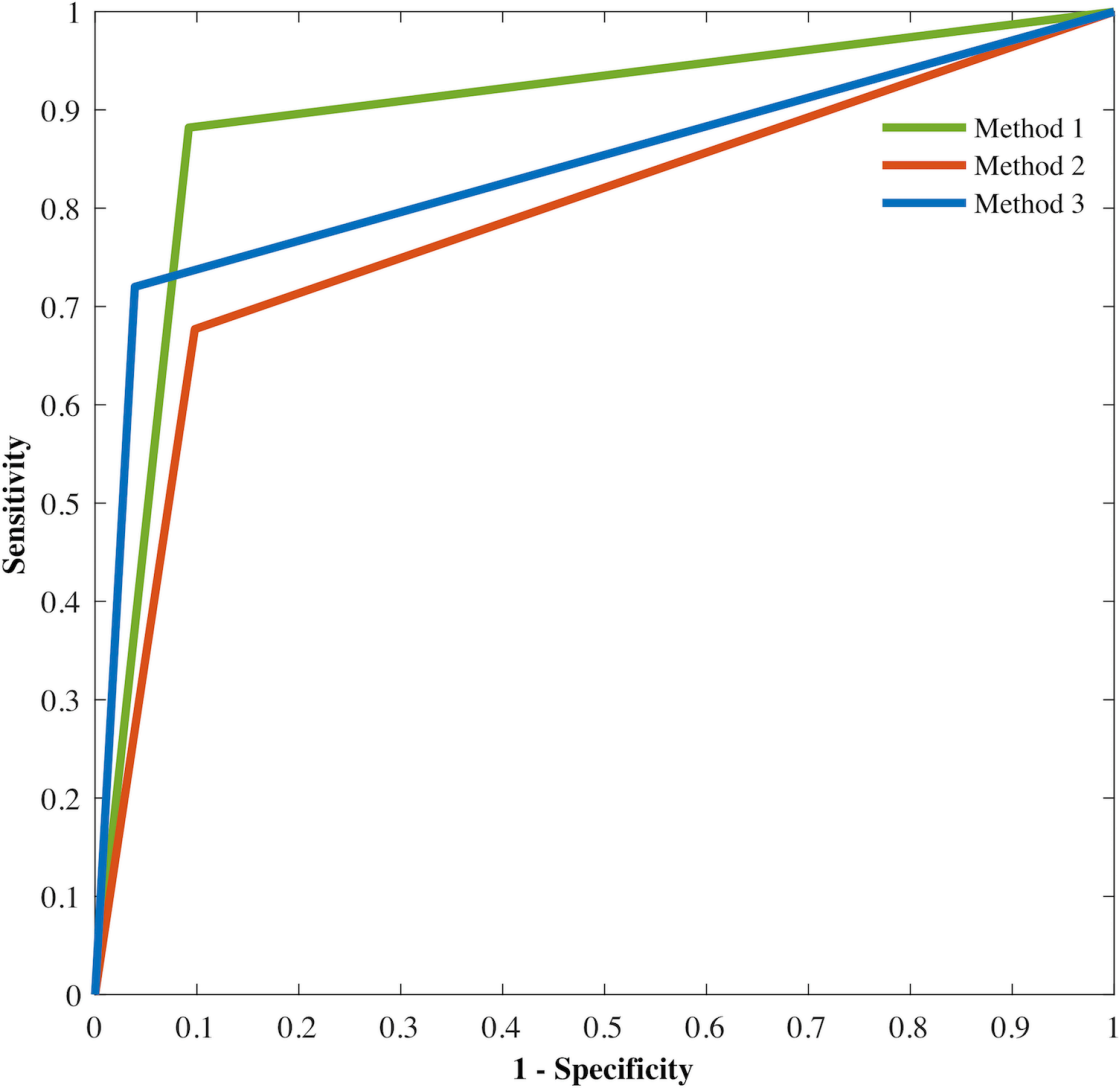
Area under the receiver operating characteristic (ROC) curve (AUC) obtained for the three methods. Method 1 reached a higher AUC, followed by method 3 and then method 2.

Sensitivity was defined as the probability for a method to detect FEs when FEs occurred, and specificity as the probability for a method to detect normal movement when FEs did not occur. Table 4 summarizes the specificity and sensitivity obtained by each of the three methods in discriminating a FE from normal movement. M1 showed to better detect FEs when they were present with a sensitivity of 88.2% compared to M2 (67.7%) and M3 (72%). As for the specificity, the three methods showed a high probability (above 90%) to detect normal movement where there was not FEs, being M3 the highest with a 96.1%. The ROC results for all methods are provided in Fig 2. Based on the area under the ROC curve (AUC), M1 better discriminated FEs from normal movement (0.895) compared to M2 and M3 (0.790 and 0.840, respectively) (Fig 2).

**Table 4:** Receiver operating characteristic (ROC) analysis for identifying freezing episodes in patients with Parkinson’s disease by three different methods.

Method	AUC	Sensitivity (%)	Specificity (%)
1	.895	88.2	90.8
2	.790	67.7	90.2
3	.840	72	96.1

Note: AUC: area under the curve

## Discussion

The purpose of this study was to introduce a methodology that better discriminates FEs from normal movement in individuals with PD, compared to other methods recently described in the literature. To our knowledge, this is the first study to investigate the effectiveness of the CWT to differentiate FEs from normal movement in terms of number, duration and time of occurrence using kinematic position data. Our main findings are that the CWT is a highly sensitive tool to identify FEs in kinematic position data. Moreover, we found that a freezing criterion based on a baseline time, such as quiet stance, rather than an amplitude decrease, is more effective in identifying FEs.

The SIP task was chosen in this study to evaluate freezing in individuals with PD. Although it is well known that it is more likely that FOG occurs when initiating walking, turning, passing through narrow spaces, and/or approaching to a destination [26, 40], as well as situations where cognitive, limbic, or motor information needs to be processed [24], the SIP task has previously been shown to be successful inducing FEs in individual with PD [4, 5]. In addition, previous studies showed that freezing is not exclusively related to the process of walking but to the cyclical aspects of bilateral coordination of limb movement, suggesting that lower limb tasks other than gait could provide valuable insight into the nature of freezing in PD [1, 41]. Moreover, the SIP involves performance in a controlled space, facilitating the security of patients with PD as well as data acquisition. In our study, the sensitivity of the SIP task in inducing freezing in those participants who were screened as freezers based on the FOGQ was lower compared to that in a previous study where the same task was used (41.7% vs 87%) [4]. However, our SIP task successfully induced FEs in an important number of trials. Therefore, we estimated that our experimental approach was adequate to address the purpose of our study of defining a methodology for automatic detection of freezing.

Different attempts with mathematical functions have been made in order to objectively identify FEs in individuals with PD. A sensitivity of 73.1% and a specificity of 81.6% in FEs detection was reached in a recent study were the FFT was used with acceleration data, a method that elicits information only in the frequency domain [34]. A higher sensitivity (84.9%) and similar specificity (81.01%) was reached decomposing acceleration data obtained from IMUs with the CWT, a method that considers time-frequency components of the signal [37]. Our M1 reached higher numbers of sensitivity (88.3%) and specificity (90.7%) by using the CWT with kinematic position data compared to the recent study that similarly used a time-frequency method [37]. The different results found between Rezvanian and Lockhart study [37] and ours could be a result of: (1) the type of kinematic data analyzed, since our study deals with positions obtained from a motion tracking system while their work with accelerations obtained from IMUs, suggesting a loss of sensitivity when calculating the integral of position data; (2) the position of the IMU or marker analyzed, since position data in our study is obtained from markers placed on the heel while accelerations in their study are obtained from IMUs placed on the shanks, possibly suggesting that the closer the landmark analyzed is to the end of the limb, the more sensitive the FEs detection would be; and (3) the freezing criteria used in each study, since our algorithm is more robust not only considering a FE as small as 0.5 s but also integrating a criterion of a minimum window of 2 s of successful task performance after a FE occurs in order to overcome freezing. This is supported by the difference seen between M1 and M2, a method that differs from M1 in not taking into account the minimum window of 2 s to overcome a FE. However, the differences in the number and the total duration time of FEs between the videos and the methods were smaller for M1 compared to M2. In fact, significance was obtained for the differences in duration time between videos and methods for M1 and M2. Moreover, the trend for the differences in number of FEs might have reached significance if a less conservative correction for the post-hoc analysis had been used. It should also be noted that M1 reached better accuracy in detecting the time of occurrence of FE and higher numbers of sensitivity and specificity when discriminating freezing from normal movement compared to M2.

Based on the most accepted definition of FOG [26], it is suggested that FOG refers to a complete movement arrest as well as to periods with severely disrupted walking patterns [28]. This wide interpretation has allowed the use of different freezing criteria that are captured in the same definition to detect FEs not only for lower limb but also for upper limb, such as no effective movement [4, 5, 9, 10, 12], amplitude decreases [18, 42], cycle latencies [2, 3, 31], or a combination of two or more [8, 11, 33]. Our methods 1 and 3 (M1 and M3, respectively) are distinct from each other in that the first one defines the threshold to identify FEs based on baseline (i.e. no movement during a 5 s quiet stance period) while the second one does it based on amplitude decreases. Although we did not find significant pairwise effects in the differences of number and total duration of FEs between the videos and these two methods, M1 was closer to the numbers obtained from the videos. The significant differences found in the accuracy to detect the time of occurrence of FEs between M1 and M3, in addition to the higher values obtained for M1 in the AUC suggests baseline to be more efficient and trustworthy when discriminating freezing from normal movement rather than amplitude, which might be capturing other parkinsonian gait characteristics, such as festination and shuffling steps.

There is a growing interest for continuous monitoring of mobility in PD as it could allow to better understand the activities triggering FOG and design personalized interventions. Continuous assessment of mobility has only recently become a viable solution due to technological development of wearable devices and algorithms to characterize and quantify, in real-time, gait movements and FEs [34, 37]. Some methods showed good sensitivity (84.9%) and specificity (81.01%) in identifying freezing and discriminating it from normal movement [37]. However, effort has to be made to improve algorithms for detection of FOG. While our study was performed in the laboratory rather than in real life activities, we believe that the results obtained provide novel insight into ways to improve the automatic detection of freezing. This lends support to choosing freezing criteria based on baseline for further studies aiming to investigate and monitor freezing during daily life tasks.

## Conclusion

In summary, results from this study suggest that the freezing criteria used to identify an episode might influence the occurrence of FE. For instance, freezing criteria based on baseline seem to better capture complete movement arrests in individuals with PD that experience freezing, while freezing criteria based on amplitude decreases also capture abnormal gait patterns. Therefore, our findings bring into question the validity of comparisons across studies that used different freezing criteria. Moreover, the complexity of a FE requires the computation of more robust algorithms that consider the time and frequency components of the signal for better discrimination of a FE from normal movement. Further studies are needed to provide insight and confirm whether the CWT approach (1) is more sensitive when used with position data compared to acceleration data and (2) is equally as sensitive identifying freezing in other gait tasks as it is in the SIP task.
